# Promising therapeutic effects of high-frequency repetitive transcranial magnetic stimulation (HF-rTMS) in addressing autism spectrum disorder induced by valproic acid

**DOI:** 10.3389/fnins.2024.1385488

**Published:** 2024-08-22

**Authors:** Masoud Afshari, Shahriar Gharibzadeh, Hamidreza Pouretemad, Mehrdad Roghani

**Affiliations:** ^1^Department of Cognitive Psychology, Institute for Cognitive and Brain Sciences, Shahid Beheshti University, Tehran, Iran; ^2^Neurophysiology Research Center, Shahed University, Tehran, Iran

**Keywords:** autism spectrum disorder, high-frequency transcranial magnetic stimulation, valproic acid, oxidative stress, dendritic spine density

## Abstract

**Introduction:**

Autism spectrum disorder (ASD) is a neurodevelopmental condition that affects various regions of the brain. Repetitive transcranial magnetic stimulation (rTMS) is a safe and non-invasive method utilized for stimulating different brain areas. Our objective is to alleviate ASD symptoms using high-frequency rTMS (HF-rTMS) in a rat model of ASD induced by valproic acid (VPA).

**Methods:**

In this investigation, we applied HF-rTMS for ASD treatment, focusing on the hippocampus. Behavioral assessments encompassed core ASD behaviors, as well as memory and recognition tests, alongside evaluations of anxiety and stress coping strategies. Additionally, we analyzed oxidative stress and a related inflammation marker, as well as other biochemical components. We assessed brain-derived neurotrophic factor (BDNF), Microtubule-associated protein-2 (MAP-2), and synaptophysin (SYN). Finally, we examined dendritic spine density in the CA1 area of the hippocampus.

**Results:**

The results demonstrated that HF-rTMS successfully mitigated ASD symptoms, reducing oxidative stress and improving various biochemical factors, along with an increase in dendritic spine density.

**Discussion:**

Collectively, our data suggests that HF-rTMS may effectively alleviate ASD symptoms. These findings could be valuable in clinical research and contribute to a better understanding of the mechanisms underlying ASD.

## 1 Introduction

Autism spectrum disorder (ASD) is a complex neurodevelopmental condition that affects social communication and behavior (Hodges et al., 2020). While the precise cause of ASD remains unknown, it is believed that both genetic and environmental factors contribute to its development (Almandil et al., 2019).

Initially introduced as an antiepileptic drug, valproic acid (VPA) is now also used to treat neurological disorders such as bipolar disorder and migraines (Romoli et al., 2019). However, it has been identified as a teratogenic drug, associated with risks such as congenital malformations, developmental delays, reduced cognitive function, and an increased risk of ASD when taken during pregnancy (Christensen et al., 2013; Juliandi et al., 2015). Evidence also shows that VPA exposure during pregnancy in humans increases the risk of autism in children (Roullet et al., 2013; Taleb et al., 2021). Also, is commonly used to induce autistic-like behavior in rodent models for research purposes (Nicolini and Fahnestock, 2018).

ASD affects various brain regions, with the hippocampus being a crucial part impacted by the disorder (Banker et al., 2021). Studies have shown that prenatal exposure to VPA leads to oxidative stress in the hippocampus (Jian et al., 2022), potentially affecting neural structures, especially dendrites and their spines, which are essential for proper hippocampal function (Martínez-Cerdeño et al., 2016). Oxidative stress may also influence beta-secretase 1 (BACE1), which has been linked to cognitive decline (Mouton-Liger et al., 2012; Zuliani et al., 2021). Inflammation has also been reported to play an important role in the pathophysiology of ASD, with markers like TNFα increased in both ASD patients and VPA-exposed rodents (Deckmann et al., 2018). One consequence of these changes may be a reduction in the density of dendritic spines in areas such as the hippocampus, eventually leading to behavioral alterations such as core autism behaviors and cognitive declines. Additionally, there may be an increased chance of apoptosis in the hippocampus, as seen in other studies (Zohny et al., 2023).

Transcranial magnetic stimulation (TMS) is known for its ability to stimulate the brain cortex and induce changes in activity (Afshari et al., 2023). Repeated TMS (rTMS) protocols involve multiple magnetic stimulations at known frequencies over multiple sessions to produce long-term changes in brain cortex activity (Marder et al., 2022). High-frequency rTMS (HF-rTMS), typically defined as frequencies ≥ 5 Hz, can increase activity, while low-frequency rTMS (LF-rTMS) inhibits brain cortex activity (Wang J. et al., 2020). Several studies have reported the antioxidant activity of rTMS, leading to neuroprotective effects, and its ability to increase brain-derived neurotrophic factor (BDNF) (Shang et al., 2016; Medina-Fernández et al., 2018), potentially influencing dendrite growth and spine maturation. It has also been shown that TMS, specifically LF-rTMS, can have beneficial effects on models of ASD induced by both VPA and maternal separation (Tan et al., 2018; Xu et al., 2024). Also, In our previous study, we observed beneficial effects of LF-rTMS on ASD conditions, consistent with the findings of the abovementioned studies (Afshari et al., 2024). There are reports indicating that HF-rTMS does not have a treatment effect on social dysfunction in FMR^–/–^ mice (Hou et al., 2021) However, several studies report beneficial effects in different rodent models (Choung et al., 2021; Kim et al., 2024; Zhang et al., 2024). As noted, the effects of LF-rTMS and HF-rTMS on the brain differ and have been reported with varying outcomes, making it necessary to investigate both frequencies to determine the most effective method.

As mentioned earlier, there are studies that have focused on the effect of LF-rTMS on ASD, but the effects of HF-rTMS on this condition have not been thoroughly investigated. Additionally, the exact mechanisms by which TMS affects ASD models remain unknown. Therefore, the primary objective of our study is to utilize HF-rTMS treatment with a focus on the hippocampus to investigate the potential treatment effects of this method on autistic behavior and cognitive impairment in VPA-induced ASD, as well as the possible mechanisms underlying the effects of HF-rTMS.

## 2 Material and methods

### 2.1 Experimental design and animal procedure

This research study adhered to the guidelines outlined in the National Institutes of Health’s Guide for the Care and Use of Laboratory Animals, along with ethical guidelines published by Shahid Beheshti University (Approval ID: IR.SBU.REC.1401.108). Adult Wistar rats were used in the study and were maintained under standard conditions in the animal facility. The facility was kept at a temperature of around 22 ± 1°C, humidity at 45 ± 3%, and maintained a 12-h light and dark cycle. Throughout the experiment, rats had free access to standard food and clean water.

For mating purposes, each couple was placed in one cage with appropriate nesting materials. Regular checks were conducted to identify a white sperm plug on the vagina or cage, confirming successful mating. After 12.5 days of confirmed mating, one group received a single intraperitoneal injection of VPA (Daroupakhsh, Iran) prepared in saline (600 mg/kg) for ASD induction, while the other group received saline as a vehicle (Zahedi et al., 2023). Following the injection, the rats were returned to their cages.

**FIGURE 1:**
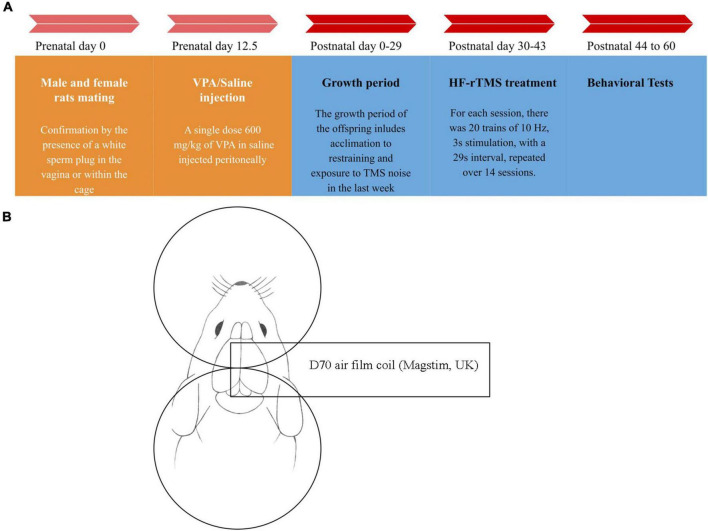
Timeline chart of the study and stimulation method diagram. **(A)** At day 12.5 after mating, valproic acid (VPA)/saline was injected into female rats. From postnatal day 30 to day 43, 14 sessions of high-frequency repeated transcranial magnetic stimulation (HF-rTMS)/Sham rTMS were applied to the rats. Behavioral studies commenced 24 h after the treatment finished. On day 60, the rats were sacrificed, and their brains were removed for further analysis. **(B)** For the stimulation, after properly restraining the rat, the center of the coil was positioned between the rat’s eye and ear, in the middle of the head with the handle perpendicular to the body. For the sham group, the coil was positioned upside down at a sufficient distance from the head to ensure the rats heard the sound produced by the device but did not receive any magnetic pulses.

After 21 days from birth (Tan et al., 2018), male offspring rats were randomly assigned to experimental groups. The study comprised four experimental groups: Sham, High-Frequency rTMS+Healthy rat (HF+H), VPA, and VPA+High-Frequency rTMS (VPA+HF) (Figure 1). For behavioral analysis (*n* = 8), biochemical analysis (*n* = 5), and histological analysis (*n* = 6), each brain hemisphere of the rats was randomly chosen for biochemical and histological analysis.

### 2.2 High-frequency repeated transcranial magnetic stimulation

The rTMS treatment protocol was adapted from a previous study with some modifications (Tan et al., 2018). This study employed high-frequency stimulation (10 Hz) with 20 trains, 30 pulses per train, and 29-s intervals, totaling 600 pulses per session for 14 consecutive days. Stimulation intensity was determined based on a preliminary experiment involving five healthy adult rats. The precise stimulation intensity was established using the stimulation threshold for forelimb movement in the rats, set at 50% of the device’s maximum stimulation output. A 70-mm figure-of-eight coil (D70 air film coil) and a Super Rapid 2 TMS device (MagStim, UK) were utilized for brain stimulation. For the treatment, the rats were restrained using a modified version of a previously established method (Weiler et al., 2021). Medical gloves were used to create sacks with the tips cut off to expose the stimulation area of the head, nose, and mouth. The rats were then secured with self-adhesive elastic wrap to form a specialized restraint setup. After fixation, no breathing struggles were observed. The restrained rats were placed on a wooden box on a table, and a coil was gently positioned on their heads, ensuring the coil surface touched the head without applying pressure. The rats were also held on the wooden box with the wrap to minimize movement. No side effects were observed from the stimulation or restraining method.

To further minimize stress and movement, the rats were acclimated by being restrained and exposed to the sound of the TMS device for one week before the main treatment. The treatment was administered in the morning between 8:00 and 12:00 PM in a low-light, quiet environment. The center of the coil was positioned on the center of the head, between the eyes and ears, with the handle perpendicular to the body (Weiler et al., 2021). For the sham group, the coil was placed upside down at a sufficient distance to ensure no magnetic pulses were delivered, while maintaining the sound level equivalent to that of the treatment group.

### 2.3 Behavioral assessment

To assess the effectiveness of the treatment, several behavioral tests were conducted 24 h after the treatment ended. These tests included the three-chamber test, self-grooming behavior, marble burying test, elevated plus maze test, Barnes maze test, novel object recognition test, and forced swim test (The test starts 24 h after treatment, and each test is performed on a separate day to ensure that rats have enough time to rest).

#### 2.3.1 Three chamber test

In accordance with the reference study (Mansouri et al., 2021), we employed the three-chamber test to assess the social interaction of rats. The test comprises three stages:

Habituation: Rats were placed in the center of the apparatus and allowed to explore freely for 10 min.

Sociability Responses: An empty metal cage was placed on one side of the apparatus, and another cage with an unfamiliar rat was placed on the opposite side. The test animal had 10 min to explore the apparatus, assessing sociability responses. The index is calculated by subtracting the time spent in the non-social stimuli chamber from the time spent in the social chamber, divided by the sum of the time spent in both the social and non-social chambers.

Direct social interaction: The time rats spent interacting with social and non-social stimuli was recorded (sniffing). The index was calculated in the same manner as the sociability index, except sniffing time was used for calculation.

#### 2.3.2 Open field test

In this test, rats were placed in a box (40 cm × 40 cm × 40 cm) for 15 min. The time spent on self-grooming behavior and the distance traveled in the box were assessed (Mansouri et al., 2021).

#### 2.3.3 Marble burying test

To assess marble burying, an apparatus (40 cm × 40 cm × 40 cm) filled with nesting material and 20 marbles arranged in 5 rows was used. Rats were placed in the apparatus for 30 min to freely explore, and the number of buried marbles was counted, representing repetitive digging behavior (Mansouri et al., 2021).

#### 2.3.4 Elevated plus maze test

The plus maze, consisting of four arms (two open and two closed) with dimensions of 50 cm × 10 cm × 40 cm, and a central platform measuring 10 cm × 10 cm, was used to evaluate rat behavior. The study focused on two behaviors: time spent in open arms and total number of entries in open arms (Servadio et al., 2016).

#### 2.3.5 Barnes maze test

The Barnes Maze task evaluated spatial learning and memory using a circular platform with 20 holes and a diameter of 120 cm. The task comprised three stages, including habituation and training trials, with parameters monitored such as latency, errors, search strategy, and total exploration of the holes in the apparatus (Zappa Villar et al., 2018).

The behavioral parameters monitored during the Barnes Maze test included: Latency: The time spent to reach the escape hole for the first time in the probe trial. Errors: The number of errors made in finding the escape hole for the first time in the probe test. Search Strategy: The strategy employed to find the escape hole, categorized into three strategies: spatial (given a score of 1), serial (given a score of 2), or random (given a score of 3). Total Exploration: The overall exploration of the holes in the apparatus.

#### 2.3.6 Novel object recognition test

This test involved a box with dimensions of 40 cm × 40 cm × 40 cm. Rats familiarized themselves with the empty arena for 5 min. On the following day, two identical objects were placed near two adjacent corners, and rats explored them for 10 min. On the third day, one object was replaced with a new one, and the rats explored for 10 min. The discrimination index was calculated to indicate the response to the new object (Zahedi et al., 2023). The index is calculated by subtracting the time spent interacting with the familiar object from the time spent with the unfamiliar object, and then dividing this difference by the sum of the two mentioned times.

#### 2.3.7 Forced swim test

A cylindrical apparatus (diameter: 30 cm, height: 50 cm) filled with clean water (25 ± 1 °C) was used for the Forced Swim Test. The habituation phase lasted for 2 min, and the test phase lasted for 5 min. Immobility time and climbing time were assessed during the test (Hu et al., 2017).

### 2.4 Biochemical assessment

After the behavioral assessment, at postnatal day 60, the rats were sacrificed under ketamine/xylazine anesthesia, and then the brain was removed for further analysis. Subsequently, the hippocampus from one brain hemisphere was homogenized in lysis buffer for further analysis.

#### 2.4.1 Reactive oxygen species (ROS) assessment

Dichlorofluorescein diacetate (DCF-DA) served as a probe for evaluating ROS. Measurements were conducted at 488 nm excitation and 525 nm emission wavelengths (Nazari-Serenjeh et al., 2024).

#### 2.4.2 Malondialdehyde (MDA) assessment

This process involved mixing the supernatant with trichloroacetic acid and TBARS reagent, followed by incubation at a temperature of 90°C for 80 min. After cooling, the samples were centrifuged at a force of 1,000 g for 10 min, and the absorbance of the supernatant was measured at a wavelength of 532 nm. The final results were expressed in ng/mg protein (Alizadeh Makvandi et al., 2021).

#### 2.4.3 Glutathione (GSH) assessment

To measure GSH levels, the Ellman reagent (DTNB) (Merck, Germany) was utilized. the measurement was taken at 412 nm. The results were expressed in ng/mg protein (Alizadeh Makvandi et al., 2021).

#### 2.4.4 Superoxide dismutase (SOD) assessment

For the assessment of SOD activity, a previously published method based on the xanthine and xanthine oxidase reaction was used, and the measurement was taken at 550 nm (Alizadeh Makvandi et al., 2021).

#### 2.4.5 Catalase (CAT) assessment

The amount of breakdown of H2O2 with CAT is the basis of this assessment, following the referenced study (Nazari-Serenjeh et al., 2024). CAT activity was then expressed as unit/mg protein.

#### 2.4.6 Tumor necrosis factor alpha (TNFα) assessment

The levels of TNFα were assessed utilizing a kit obtained from Bio-Techne, USA. The analysis employed a sandwich enzyme-linked immunosorbent assay (ELISA) method, in accordance with the manufacturer’s guidelines (Zahedi et al., 2023).

#### 2.4.7 Caspase-3 assessment

The hydrolytic activity of caspase-3 on p-nitroaniline (pNA) was utilized for this assessment, following a previously established method (Khaleghi-Mehr et al., 2023).

#### 2.4.8 Mitochondrial membrane potential (MMP) assessment

Measurement of mitochondrial membrane potential employed the fluorescent cationic dye rhodamine 123 (Keshavarz-Bahaghighat et al., 2018).

#### 2.4.9 Beta secretase 1 (BACE1) assessment

The hydrolysis of DL-BAPNA per hour (ΔA/h) served as a marker for estimating BACE1 activity in the supernatant. The protocol was based on a previously published method (Ogunsuyi et al., 2020).

#### 2.4.10 Brain-derived neurotrophic factor (BDNF) assessment

A commercial BDNF kit (RAB 1138, Sigma, USA) was used for this assessment, following the manufacturer’s instructions (Mansouri et al., 2021).

#### 2.4.11 Synaptophysin (SYN) assessment

An ELISA kit (CSB-E13827r) based on the sandwich enzyme method was used for SYN assessment. The final reaction was read on a microplate reader at a wavelength of 450 nm (Hacioglu et al., 2021).

#### 2.4.12 Microtubule-associated protein 2 (MAP2)

Assessment MAP2 assessment was based on the sandwich ELISA method, as described in a previous study (Papa et al., 2018).

#### 2.4.13 Protein content of the hippocampus

The BCA (bicinchoninic acid) method was employed to determine the protein content of the hippocampus (Smith et al., 1985).

### 2.5 Golgi staining

Following a previously established method, brain blocks were fixed in a solution containing mercury chloride (1%), potassium chromate (0.8%), potassium dichromate (1%), potassium tungstate (0.5%) for two weeks, and subsequently immersed in another solution containing lithium hydroxide (1%) and potassium nitrate (15%) (Torabi et al., 2019). For this analysis, four representative sections and four CA1 pyramidal neurons/sections were randomly selected along the dorsoventral axis at bregma coordinates from −3.2 mm up to −3.8 mm and the number of spines was counted for a length of 20 μm for second order dendrites and exclusion of primary dendrites as previously described (Mahmmoud et al., 2015; Schartz et al., 2016).

### 2.6 Statistical analysis

In analyzing the outcomes (GraphPad Prism, v9.5.1), we assessed normality visually using Q-Q plots generated from the dataset and also conducted the Brown-Forsythe test to assess equality of variance. Statistical analysis included a Two-Way ANOVA followed by a Tukey test for multiple comparisons. For examining the EPM number of entries in open arms and Barnes maze search strategy, exploration numbers and the number of errors, a Kruskal-Wallis test was performed, followed by Dunn’s test for post-hoc comparisons. Results are presented as mean ± SD, with statistical significance set at *p* < 0.05.

## 3 Results

### 3.1 Behavioral analysis

#### 3.1.1 HF-rTMS treatment alleviated social impairment in VPA-exposed rats in the three-chamber test

**FIGURE 2:**
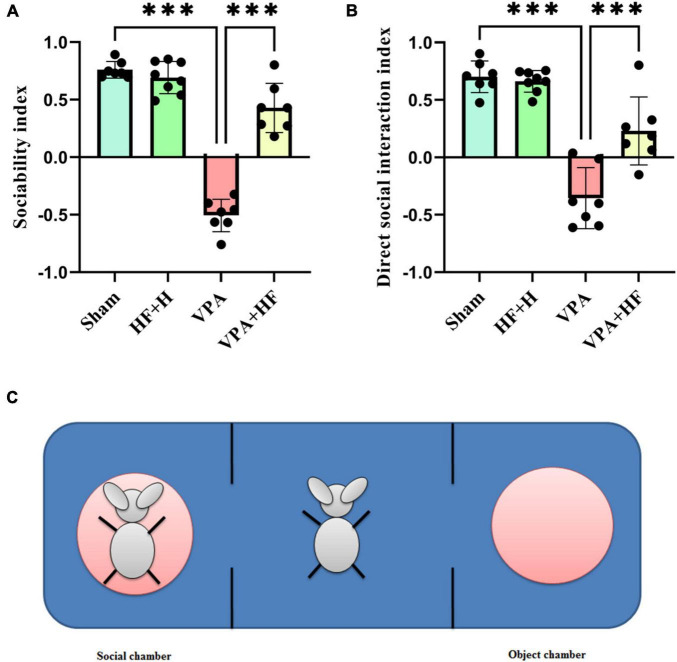
The effect of high-frequency repeated transcranial magnetic stimulation (HF-rTMS) on three chamber test of VPA-exposed rats. **(A)** Sociability index: calculated by subtracting the time spent in the non-social chamber from the time spent in the social chamber, divided by the sum of these times. **(B)** Direct social interaction index: calculated by subtracting the interaction time with non-social stimuli from the interaction time with social stimuli, divided by the sum of these times. **(C)** This illustration shows the apparatus. The rat shown on the left side is in the metal cage, referred to as the social chamber. The middle chamber contains the rat subjected to behavioral testing, and the right chamber is the object chamber with an empty, similar metal cage (Data presented as mean ± SD, *n* = 8). ***p < 0.001.

Social preference was assessed using the sociability index, which indicated a significant difference among groups [*F*(3, 18) = 96.81, *p* < 0.001, Figure 2A]. *Post hoc* analysis revealed a significant reduction in the sociability index in the VPA-exposed group compared to the sham group (*p* < 0.001), with HF-rTMS treatment showing a significant reversal of this reduction (*p* < 0.001). For direct social interaction, analysis indicated significant differences among groups [*F*(3, 18) = 30.62, *p* < 0.001, Figure 2B]. Prenatal VPA exposure reduced direct social interaction compared to the sham group (*p* < 0.001), and HF-rTMS treatment significantly improved direct social interaction compared to the VPA-exposed group (*p* < 0.001). Figure 2C illustrates this test.

#### 3.1.2 HF-rTMS treatment alleviated self-grooming behavior and distance traveled in VPA-exposed rats in the open field test

**FIGURE 3:**
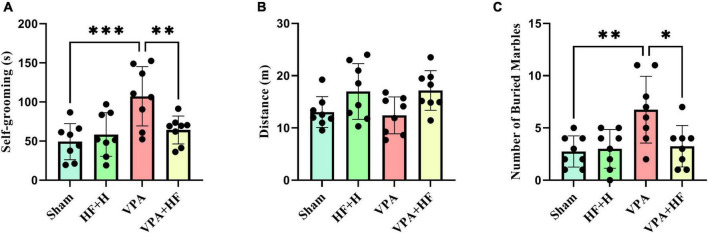
The effect of high-frequency repeated transcranial magnetic stimulation (HF-rTMS) on open field test and marble burying test of VPA-exposed rats. **(A)** Self-grooming time: calculated as the total time the rats spend grooming themselves. **(B)** Distance traveled: the total distance covered by the rats in the apparatus. **(C)** Number of buried marbles: the total count of marbles buried in the nesting materials (Data presented as mean ± SD, *n* = 8). *p < 0.05, **p < 0.01, ***p < 0.001.

Two behaviors were assessed in the open field test. Self-grooming behavior showed significant differences among groups [*F*(3, 21) = 9.226, *p* < 0.001, Figure 3A]. Rats exposed to VPA exhibited significantly more self-grooming behavior compared to the sham group (*p* < 0.001), and HF-rTMS treatment significantly reduced self-grooming behavior (*p* < 0.01). Also there were no significant changes in distance traveled among groups (Figure 3B).

#### 3.1.3 HF-rTMS treatment alleviated repetitive digging behavior in VPA-exposed rats in the marble burying test

The marble burying test, assessing repetitive digging behavior, revealed significant differences among groups [*F*(3, 21) = 5.823, *p* < 0.01, Figure 3C]. Post-hoc analysis indicated that VPA-exposed rats exhibited a significant preference for digging marbles (*p* < 0.01), and HF-rTMS treatment significantly reduced this behavior (*p* < 0.05).

#### 3.1.4 HF-rTMS treatment alleviated anxiety behavior in VPA-exposed rats in the elevated plus maze test

**FIGURE 4:**
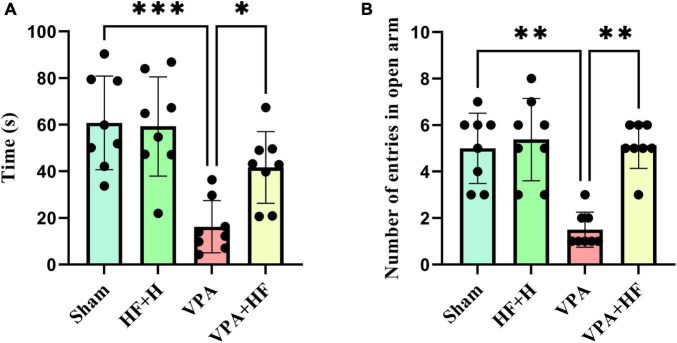
The effect of high-frequency repeated transcranial magnetic stimulation (HF-rTMS) on elevated plus maze test of VPA-exposed rats. **(A)** Time spent in open arms: Represents anxiety levels; decreased time indicates higher anxiety. **(B)** Number of entries to open arms: fewer entries suggest increased anxiety (Data presented as mean ± SD, *n* = 8). *p < 0.05, **p < 0.01, ***p < 0.001.

Anxiety was assessed using the elevated plus maze, where more time spent in the open arms indicates lower anxiety. Analysis revealed significant differences among groups [*F*(3, 21) = 12.88, *p* < 0.001, Figure 4A]. The VPA exposure group spent significantly less time in open arms (*p* < 0.001), and HF-rTMS treatment increased this time significantly compared to the VPA exposed group (*p* < 0.05). The number of times rats entered open arms also showed significant differences among groups (Figure 4B). The VPA exposed group significantly preferred fewer entries into open arms (*p* < 0.01), and HF-rTMS significantly reversed this behavior (*p* < 0.01).

#### 3.1.5 HF-rTMS treatment improved spatial memory in VPA-exposed rats in the Barnes maze test

**FIGURE 5:**
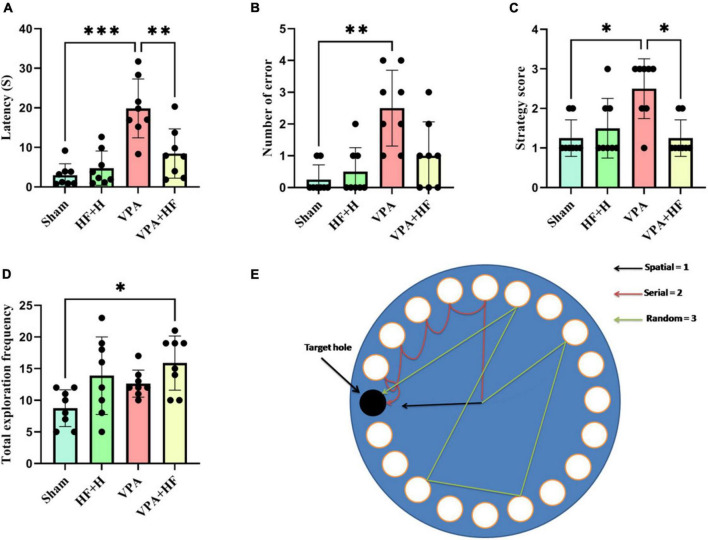
The effect of high-frequency repeated transcranial magnetic stimulation (HF-rTMS) on Barnes maze test of VPA-exposed rats. **(A)** Time spent for the first reach in the target hole. **(B)** Number of errors until finding the target hole. **(C)** Strategy used for finding the target hole. **(D)** Total frequency of exploration during the probe test. **(E)** The picture depicts the apparatus and illustrates how the search strategy was calculated (Data presented as mean ± SD, *n* = 8). *p < 0.05, **p < 0.01, ***p < 0.001.

The Barnes maze test, assessing spatial memory, showed significant differences in the time rats spent reaching the former escape hole in the probe trial [*F*(3, 21) = 13.21, *p* < 0.001, Figure 5A]. The VPA exposed group took significantly more time compared to the sham group (*p* < 0.001), and HF-rTMS treatment reduced this time compared to untreated rats (*p* < 0.01). Significant differences were observed in the number of errors to find the escape hole among groups (Figure 5B), VPA rats exhibited a significantly increased number of errors in finding the escape hole (*p* < 0.01). Strategy analysis indicated that VPA-exposed rats significantly preferred a random strategy (*p* < 0.05), and HF-rTMS shifted this preference toward a spatial strategy (*p* < 0.05) (Figure 5C). Total number of exploration during the probe trial showed significant differences among groups (Figure 5D), with significant changes observed between the Sham and VPA+HF groups (*p* < 0.05). Figure 5E illustrates how the search strategy was calculated.

#### 3.1.6 HF-rTMS treatment improved recognition memory in VPA-exposed rats in the novel object recognition test

**FIGURE 6:**
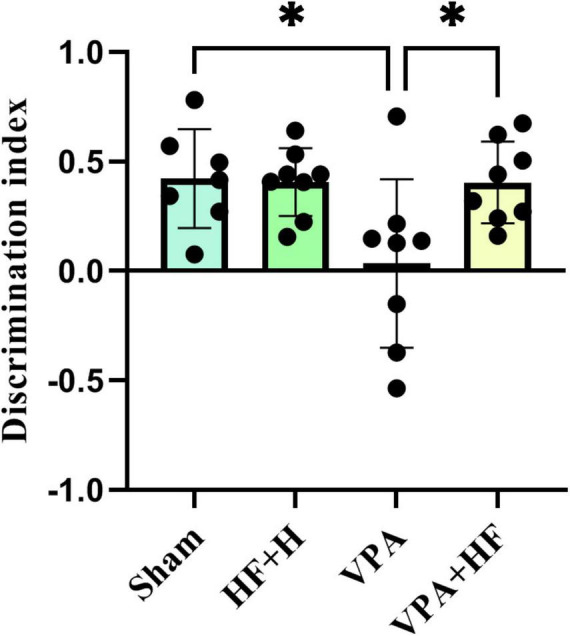
The effect of high-frequency repeated transcranial magnetic stimulation (HF-rTMS) on novel object recognition test of VPA-exposed rats. Discrimination index between new and old objects in the apparatus: calculated by subtracting the interaction time of the rat with the old object from the interaction time with the new object, divided by the sum of these times (Data presented as mean ± SD, *n* = 8). *p < 0.05.

The discrimination index in the novel object recognition test revealed significant differences among groups [*F*(3, 20) = 5.725, *p* < 0.01, Figure 6]. VPA-exposed rats exhibited a significantly reduced discrimination index (*p* < 0.05), and HF-rTMS significantly reversed this change compared to VPA-exposed rats (*p* < 0.05).

#### 3.1.7 HF-rTMS treatment improved stress coping strategy in VPA-exposed rats in the forced swim test

**FIGURE 7:**
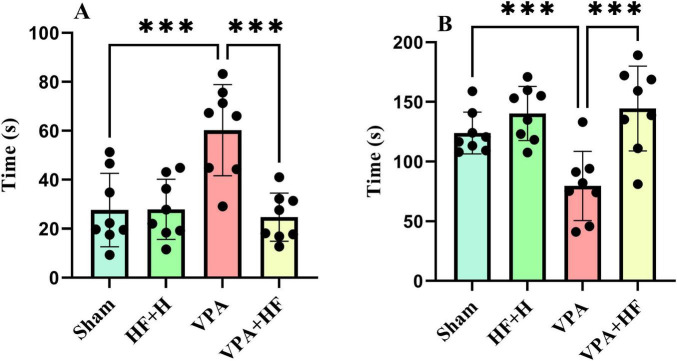
The effect of high-frequency repeated transcranial magnetic stimulation (HF-rTMS) on forced swim test of VPA-exposed rats. **(A)** Immobility time: the duration the rat remains motionless on the surface of the water. **(B)** Time spent climbing: the total time the rat spends attempting to climb up from the walls of the apparatus (Data presented as mean ± SD, *n* = 8). ***p < 0.001.

The forced swim test, evaluating stress coping strategy, showed significant differences in the time spent immobile in water [*F*(3, 21) = 10.89, *p* < 0.001, Figure 7A]. VPA-exposed rats spent significantly more time immobile (*p* < 0.001) compared to the sham group, and HF-rTMS treatment significantly reversed this change (*p* < 0.001). The time spent climbing from the apparatus also exhibited significant differences among groups [*F*(3, 21) = 36.86, *p* < 0.001, Figure 7B]. VPA-exposed rats spent significantly less time climbing (*p* < 0.001), and HF-rTMS treatment significantly increased climbing time (*p* < 0.001).

### 3.2 Biochemical parameters analysis

#### 3.2.1 HF-rTMS treatment alleviated oxidative stress in VPA-exposed rats

**FIGURE 8:**
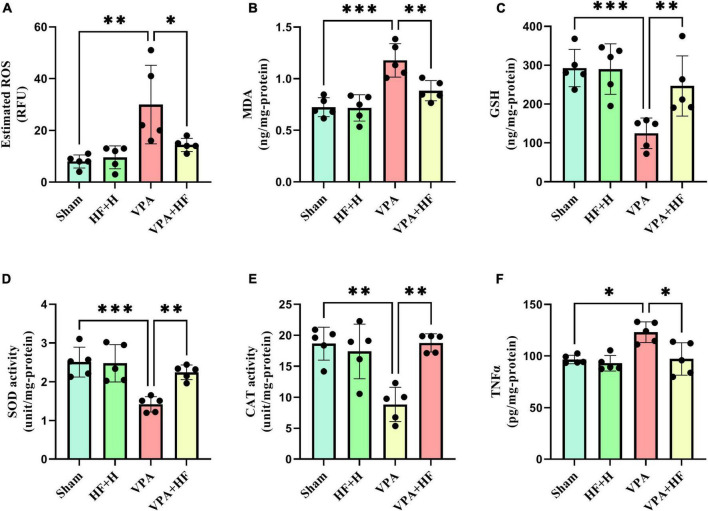
The effect of high-frequency repeated transcranial magnetic stimulation (HF-rTMS) on oxidative stress and inflammation in the hippocampus of VPA-exposed rats. **(A)** Reactive oxygen species (ROS), **(B)** Malondialdehyde (MDA), **(C)** Glutathione (GSH), **(D)** Superoxide dismutase (SOD), **(E)** Catalase (CAT), **(F)** Tumor necrosis factor alpha (TNFα) (Data presented as mean ± SD, *n* = 5). *p < 0.05, **p < 0.01, ***p < 0.001.

In the analysis of oxidative stress and inflammation (Figures 8A–F), significant differences were observed in various biochemical parameters, including ROS [*F*(3, 12) = 7.344, *p* < 0.01], MDA [*F*(3, 12) = 26.92, *p* < 0.001], GSH [*F*(3, 12) = 12.96, *p* < 0.001], SOD [*F*(3, 12) = 13.18, *p* < 0.001], CAT [*F*(3, 12) = 12.41, *p* < 0.001], and TNFα [*F*(3, 12) = 7.941, *p* < 0.01]. ROS and MDA, as oxidative factors, significantly increased with prenatal VPA exposure (*p* < 0.01 and *p* < 0.001). HF-rTMS treatment significantly reduced the levels of these oxidants (*p* < 0.05 and *p* < 0.01). In terms of antioxidant parameters (GSH, SOD, and CAT), VPA significantly reduced these parameters in the hippocampus (*p* < 0.001, *p* < 0.001, *p* < 0. 01). Rats under HF-rTMS treatment showed an improved antioxidant defense system (GSH: *p* < 0.01, SOD: *p* < 0.01, and CAT: *p* < 0.01). TNFα, as an inflammation marker, significantly increased in VPA-exposed rats compared to the sham group (*p* < 0.05), and HF-rTMS treatment significantly reduced TNFα levels compared to VPA-exposed rats (*p* < 0.05).

#### 3.2.2 Other biochemical factors

**FIGURE 9:**
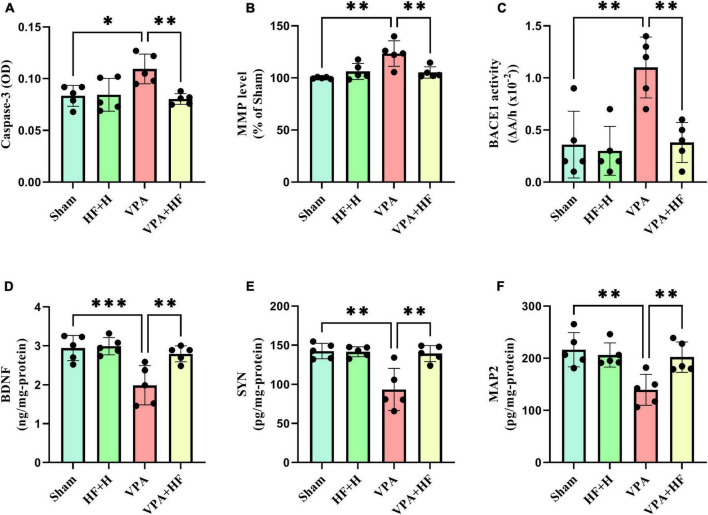
The effect of high-frequency repeated transcranial magnetic stimulation (HF-rTMS) on other biochemical factors in the hippocampus of VPA-exposed rats. **(A)** Caspase-3, **(B)** Mitochondrial membrane potential (MMP), **(C)** Beta-secretase-1 (BACE1), **(D)** Brain-derived neurotrophic factor (BDNF), **(E)** Synaptophysin (SYN), **(F)** Microtubule-associated protein 2 (MAP2) (Data presented as mean ± SD, *n* = 5). *p < 0.05, **p < 0.01, ***p < 0.001.

Significant differences among groups were observed in caspase-3 [*F*(3, 12) = 6.748, *p* < 0.01], MMP [*F*(3, 12) = 10.30, *p* < 0.01], and BACE1 [*F*(3, 12) = 11.89, *p* < 0.001]. Prenatal VPA exposure significantly increased caspase-3, MMP, and BACE1 in the hippocampus compared to the sham group (*p* < 0.05, *p* < 0.01, and *p* < 0.01, respectively). There was also a significant reduction in these factors under HF-rTMS treatment compared to non-treated VPA-exposed rats (*p* < 0.01) (Figures 9A–C).

Two-way analysis of BDNF [*F*(3, 12) = 13.63, *p* < 0.001], SYN [*F*(3, 12) = 10.56, *p* < 0.01], and MAP2 [*F*(3, 12) = 9.896, *p* < 0.01] revealed significant differences between groups. VPA-exposed rats showed significantly lower levels of BDNF (*p* < 0.001), SYN (*p* < 0.01), and MAP2 (*p* < 0.01) compared to sham groups. When comparing the VPA+HF group to the VPA exposed group, there were significant increases in these parameters (BDNF: *p* < 0.01, SYN: *p* < 0.01, and MAP2: *p* < 0.01) (Figures 9D–F).

### 3.3 HF-rTMS treatment increased dendritic spine density in VPA-exposed rats using the Golgi impregnation method

**FIGURE 10:**
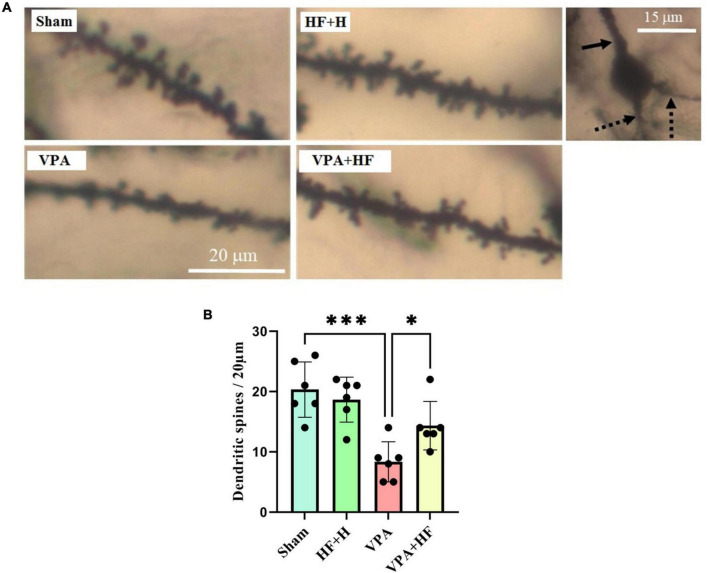
The effect of high-frequency repeated transcranial magnetic stimulation (HF-rTMS) on dendritic spine density in CA1 area of the hippocampus of VPA-exposed rats. **(A)** Representative images displaying dendritic spines from all experimental groups. The solid arrow indicates the apical dendrite, while the dotted arrows indicate the basal dendrites of a pyramidal neuron’s soma in the hippocampal CA1 area, as assessed in this study. **(B)** Quantification of dendritic spine density (Data presented as mean ± SD, *n* = 6). *p < 0.05, ***p < 0.001.

The Golgi impregnation analysis revealed significant differences between groups [*F*(3, 15) = 13.88, *p* < 0.001]. Prenatal VPA injection significantly reduced the density of spines in the CA1 region of the hippocampus compared to the sham group (*p* < 0.001). Interestingly, HF-rTMS treatment in this condition significantly increased the dendritic spine density in this area (*p* < 0.05) (Figures 10A, B).

## 4 Discussion

In this study, we evaluated the potential therapeutic impact of HF-rTMS on ASD rats induced by prenatal VPA exposure. Our findings demonstrate that prenatal VPA administration leads to long-term effects on the nervous system, influencing various behavioral characteristics. Prolonged HF-rTMS treatment can effectively reverse cognitive dysfunction associated with ASD.

ASD affects multiple brain regions, with some identified as significant contributors to ASD symptoms, including the hippocampus. Primarily involved in learning and memory, the hippocampus is susceptible to damage from various conditions (Anand and Dhikav, 2012). Consequently, hippocampal abnormalities are implicated in cognitive impairments associated with ASD (Banker et al., 2021). Animal models of ASD also reveal abnormalities in synaptic functions within the hippocampus. Moreover, hippocampus connectivity with other brain areas, such as the striatum and amygdala, may have a role in repetitive behaviors, another core symptom of ASD, as well as in social dysfunction (Kim et al., 2016; Tan et al., 2018). Furthermore, this structure plays a role in anxiety and stress management (Ghasemi et al., 2022).

In our study, prenatal injection of VPA resulted in behavioral changes in offspring. In addition to core ASD behaviors such as reduced social interaction, increased grooming, and repetitive digging behavior, we observed anxiety, impaired spatial memory, reduced recognition memory, and decreased stress coping strategies in these rats. Other studies have also reported cognitive impairments in prenatal VPA-induced autism (Nicolini and Fahnestock, 2018).

The hippocampus of offspring rats exposed prenatally to VPA injection showed increased oxidative stress and inflammation in other studies (Cristiano et al., 2022; Saadat et al., 2023). Oxidative stress has the potential to damage neural cells in the hippocampus and increase apoptosis (Saadat et al., 2023). Damage caused by oxidative stress may also increase BACE1 activity, which is related to the accumulation of amyloid β in neural cells (Mouton-Liger et al., 2012). These events may potentially lead to cognitive impairments. MMP also plays a role as a marker for cell health (Sakamuru et al., 2016). Mitochondrial dysfunction can lead to elevated ROS levels and eventually increased oxidative stress (Lenaz, 2001). Additionally, microglial activation, observed in this model through triggering receptor expressed on myeloid cells 2 downregulation (Luo et al., 2023), which has been implicated in autistic individuals (Liao et al., 2020). In our study, VPA-induced autism led to increased oxidative factors, reduced antioxidants, elevated caspase 3, and increased MMP levels, ultimately leading to heightened BACE1 activity.

BDNF is a neurotrophic component involved in neural development, neural plasticity, and synaptogenesis (Wei et al., 2020). It may also increase antioxidant activity in the body and have a protective effect against oxidative damage (Wang D. et al., 2020). BDNF may also increase MAP2 expression in neural cells (Chen et al., 2017). MAP2 is crucial in the cytoskeleton of neural cells, playing a role in microtubule polymerization and ultimately being associated with axon and dendrite structure (Chen et al., 2017). It has also been reported that the CA1 area of the hippocampus is important for social recognition in ASD (Chai et al., 2021). Evidence also shows that the CA1 area is critical in terms of cognitive dysfunction when affected by related disorders like dementia (Langdon et al., 2013). In other words, in the VPA model, when it comes to cognitive impairment, this area is also important to consider, as other studies have shown reduced dendritic spine density in pyramidal neurons of this area and spatial memory impairment (Lima-Castañeda et al., 2023). In our experiment, VPA-exposed rats showed a decrease in BDNF and MAP2, which was also evident in histological analysis with a reduction in dendritic spines in the CA1 area of the hippocampus.

Several studies have used TMS to treat individuals with ASD, but despite all efforts, TMS therapy is still in its early stages (Savino et al., 2023). One study on rats showed that LF-rTMS can improve ASD induced by neonatal isolation through the regulation of GABA transmission (Tan et al., 2018). In another study, LF-rTMS on FMR-/- mice improved social dysfunction, but they also reported that HF-rTMS cannot affect social dysfunction (Hou et al., 2021). In our study, HF-rTMS improved the core symptoms of ASD in the prenatal VPA model. HF-rTMS in preclinical research has shown the ability to improve cognitive impairment (Ma et al., 2019). It also reduced immobility time in mice (Cambiaghi et al., 2022) and anxiety in radiation induced brain injury model (Qin et al., 2024), as observed in our study with VPA-exposed rats.

Several studies on autistic patients have reported that HF-rTMS may help relieve core symptoms associated with ASD. For example, a study by Yang et al. reported that HF-rTMS on the left inferior parietal lobule can improve core symptoms of ASD in children (Yang et al., 2019). Additionally, in another study by Enticott et al. (2014) 5 Hz rTMS on the dorsomedial prefrontal cortex reduced social impairment. On the other hand, our study focused on the hippocampus. HF-rTMS has been used in several preclinical studies to reverse hippocampal dysfunction. For instance, in one study on an Alzheimer’s mouse model, HF-rTMS showed therapeutic effects and activated the dopaminergic pathway (Choung et al., 2021). In another study, also in an Alzheimer’s model, HF-rTMS demonstrated neuroprotective effects (Kim et al., 2024). Additionally, in a study comparing both LF-rTMS and HF-rTMS on Alzheimer’s disease, it was reported that both frequencies had therapeutic effects. This suggests that despite different protocols, both LF and HF-rTMS can have beneficial outcomes (Zhang et al., 2024).

Another factor that may affect rat performance in behavioral tests is mobility. Although the distance traveled in the open field test is not enough to make it significant, it seems to have an increased effect on the mobility of the rats (evident in the excessive exploration frequency in the Barnes maze test compared to sham rats). Because the coil not only stimulates the desired areas but also stimulates other parts of the brain, this outcome in rat studies may be observed. As other studies have also reported, this increased activity may be related to striatal alteration (Arfani et al., 2017). Also, we did not observe any adverse effects with HF-rTMS on rats.

Studies have claimed that rTMS has an antioxidant effect (Medina-Fernández et al., 2018). This device has the ability to reduces neural cell death induced by oxidative stress (Kim et al., 2024). Conversely, it also affects inflammatory markers, leading to a reduction in ROS production (Medina-Fernández et al., 2018). However, the exact mechanism of the antioxidant activity of rTMS remains unclear. In our study, HF-rTMS over a 14-day period reduced oxidative markers and increased antioxidant activity. It also improved MMP and TNFα, which may further reduce oxidative damage and prevent apoptosis, as reported in our study. Reduction of oxidative stress may also play a role in reducing BACE1 and, therefore, improving cognitive processes. Our data also showed an increase in BDNF in the hippocampus under HF-rTMS treatment, which may also contribute to the reduction of oxidative stress, as mentioned earlier. Another study reported increased BDNF and SYN in the hippocampus after rTMS treatment (Shang et al., 2016). As mentioned before, BDNF may also increase MAP2 (Chen et al., 2017). The increased levels of MAP2, SYN, and eventually dendritic spines in the hippocampus lead to better behavioral outcomes with HF-rTMS treatment.

One limitation of our study is the lack of detailed histological analysis, such as immunohistochemistry and other methods, which are recommended for future studies. Additionally, investigating the density of each type of dendritic spine could provide a better understanding of the effectiveness of HF-rTMS treatment, and this is also recommended for future research. While our study did not aim to explore sex differences or the treatment effects of HF-rTMS on different genders, these are important considerations for future investigations.

In conclusion, our experiment demonstrated that HF-rTMS has the potential to improve ASD symptoms induced by VPA in rats. It showed efficacy in enhancing memory, reducing anxiety, and improving stress coping strategies. These improvements may be attributed to the antioxidant properties of HF-rTMS and its capacity to increase levels of BDNF, SYN, and the density of dendritic spines.

## Data Availability

The raw data supporting the conclusions of this article will be made available by the authors, without undue reservation.
